# Supplementary material for: Evaluating metabolome-wide causal effects on risk for psychiatric and neurodegenerative disorders
Source: BMC Med. 2025 Jun 2;23:326. doi: 10.1186/s12916-025-04129-4 (PMC12131761; doi:10.1186/s12916-025-04129-4)

**Additional File 1**

**Evaluating metabolome-wide causal effects on risk for psychiatric and neurodegenerative disorders**

Lachlan Gilchrist^1,2^, Julian Mutz^1^, Pirro Hysi^3,4,5^, Cristina Legido-Quigley^6,7^, Sulev Koks^8,2^, **Cathryn M. Lewis**^1,9,^**^11^** and **Petroula Proitsi**^10,^**^11^**

^1^ Social, Genetic and Developmental Psychiatry Centre, Institute of Psychiatry, Psychology & Neuroscience, King's College London, London, United Kingdom

^2^ Perron Institute for Neurological and Translational Science, Perth, Australia

^3^ Department of Ophthalmology, King’s College London, London, UK

^4^ Department of Twins Research and Genetic Epidemiology, King’s College London, London, UK

^5^ Sørlandet Sykehus Arendal, Arendal, Norway

^6^ Systems Medicine, Institute of Pharmaceutical Science, Life Science & Medicine, King's College London, London, UK

^7^ Steno Diabetes Center Copenhagen, Copenhagen, Denmark

^8^ Centre for Molecular Medicine and Innovative Therapeutics, Murdoch University, Perth, Australia

^9^ Department of Medical and Molecular Genetics, King's College London, London, United Kingdom

^10^ Centre for Preventive Neurology, Wolfson Institute of Population Health, Queen Mary’s University of London

**^11^ equally contributing authors/co-corresponding authors: Dr. Petroula Proitsi (p.proitsi@qmul.ac.uk) and Prof. Cathryn Lewis (**[**cathryn.lewis@kcl.ac.uk**](mailto:cathryn.lewis@kcl.ac.uk)**)**

**S1: Description of genetic correlations between neuropsychiatric disorders**

Psychiatric disorders were strongly genetically correlated (*r_g_* range: 0.37-0.85, *p*-value range: 1.95 x 10^-148^ - 7.34 x 10^-66^, *p_FDR_* range: 5.46 x 10^-147^ - 3.43 x 10^-65^). This pattern was not observed between the neurodegenerative disorders, where only AD and ALS genetically correlated after FDR correction (*r_g_* [SE] = 0.34 [0.004], *p*-value = 6.75 x 10^-6^, *p_FDR_*  = 2.10 x 10^-5^). Between the psychiatric and neurodegenerative disorders, SCZ was significantly genetically correlated with AD (*r_g_* [SE] = 0.11 [0.003], *p*-value = 1.76 x 10^-3^; *p_FDR_* = 0.005). MS was genetically correlated with ANX, DEP and SCZ (*r_g_* range: 0.07- 0.13, *p*-value range: 1.48 10^-7^- 0.009, *p_FDR_* range: 5.90 x 10^-7^ – 0.02) (**Additional File 2; Supplementary Table 1**).

**Fig. S2: Heatmap of genetic correlations between neuropsychiatric disorders, calculated using linkage disequilibrium score regression (LDSC) within GenomicSEM.**

**Fig. S3: Forest plots of IVW-MR estimates for the effect of significant polygenic metabolites on unadjusted and BMI, waist-to-hip ratio and educational attainment adjusted neuropsychiatric outcomes.**


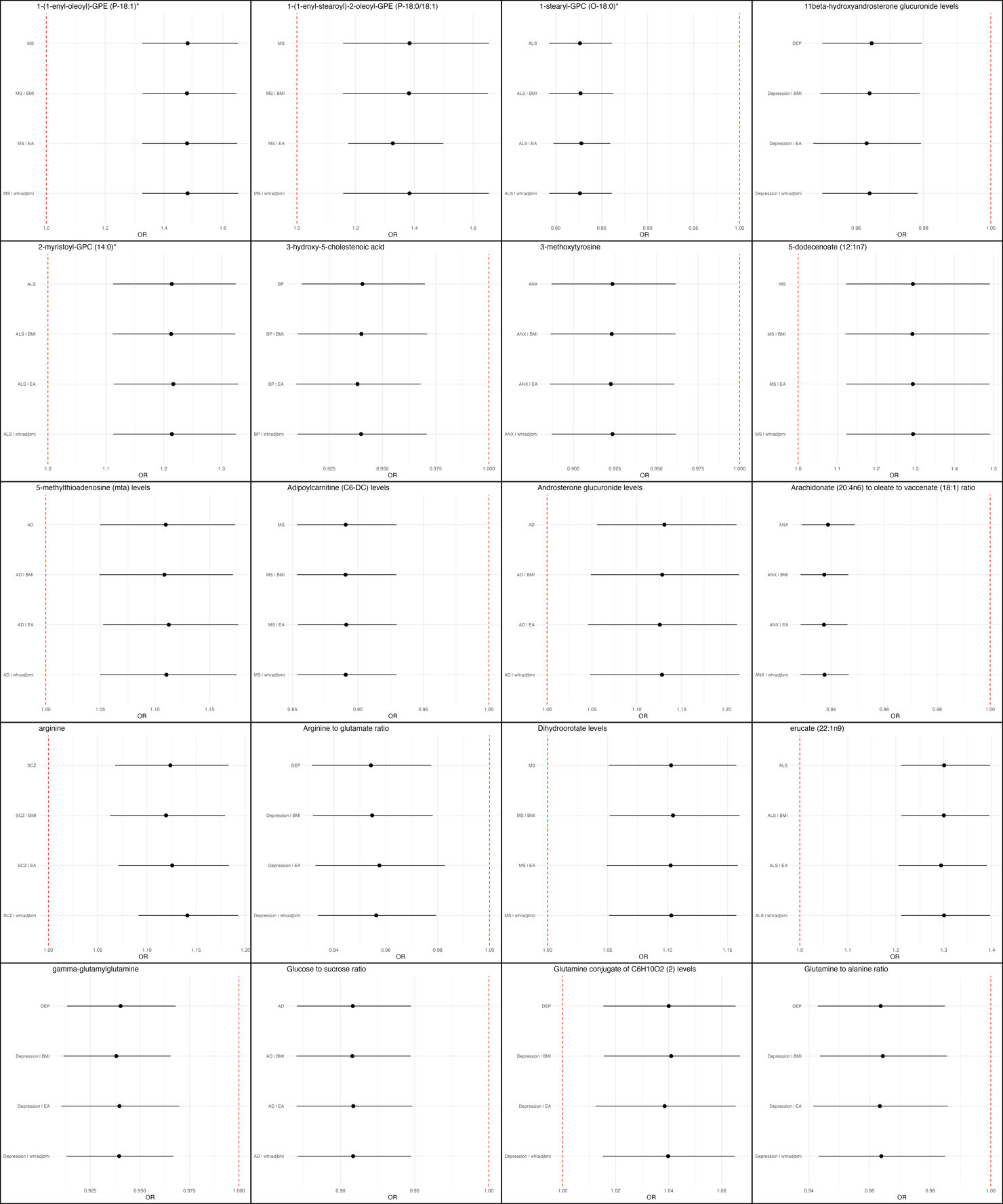


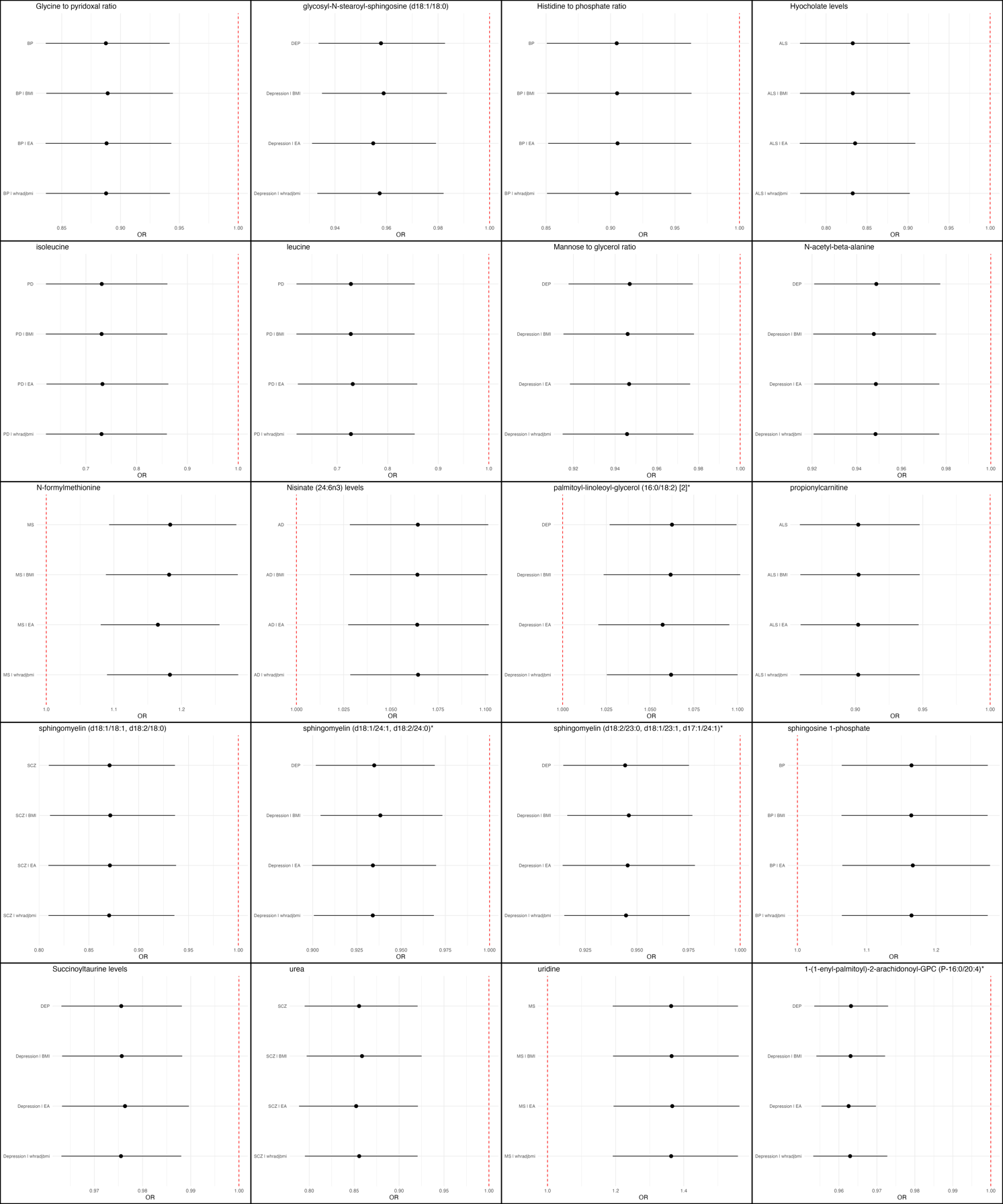


**Fig S4: Simplified metabolic pathway from linoleic acid (LA) to arachadonic acid (AA) derived from KEGG pathway hsa00591**


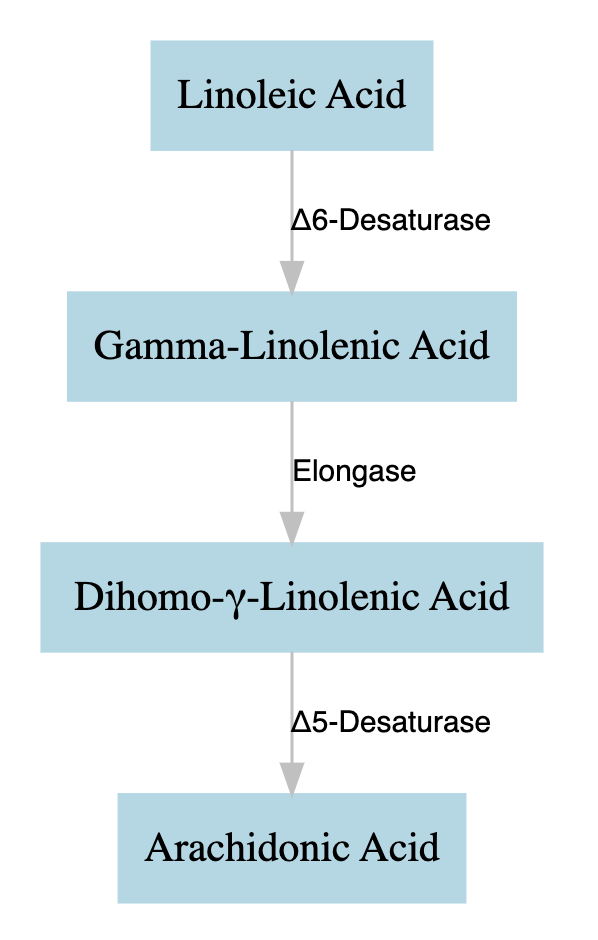


**Fig S5: Simplified metabolic pathway for sphingomyelin and ceramide metabolism from KEGG pathway hsa00600**


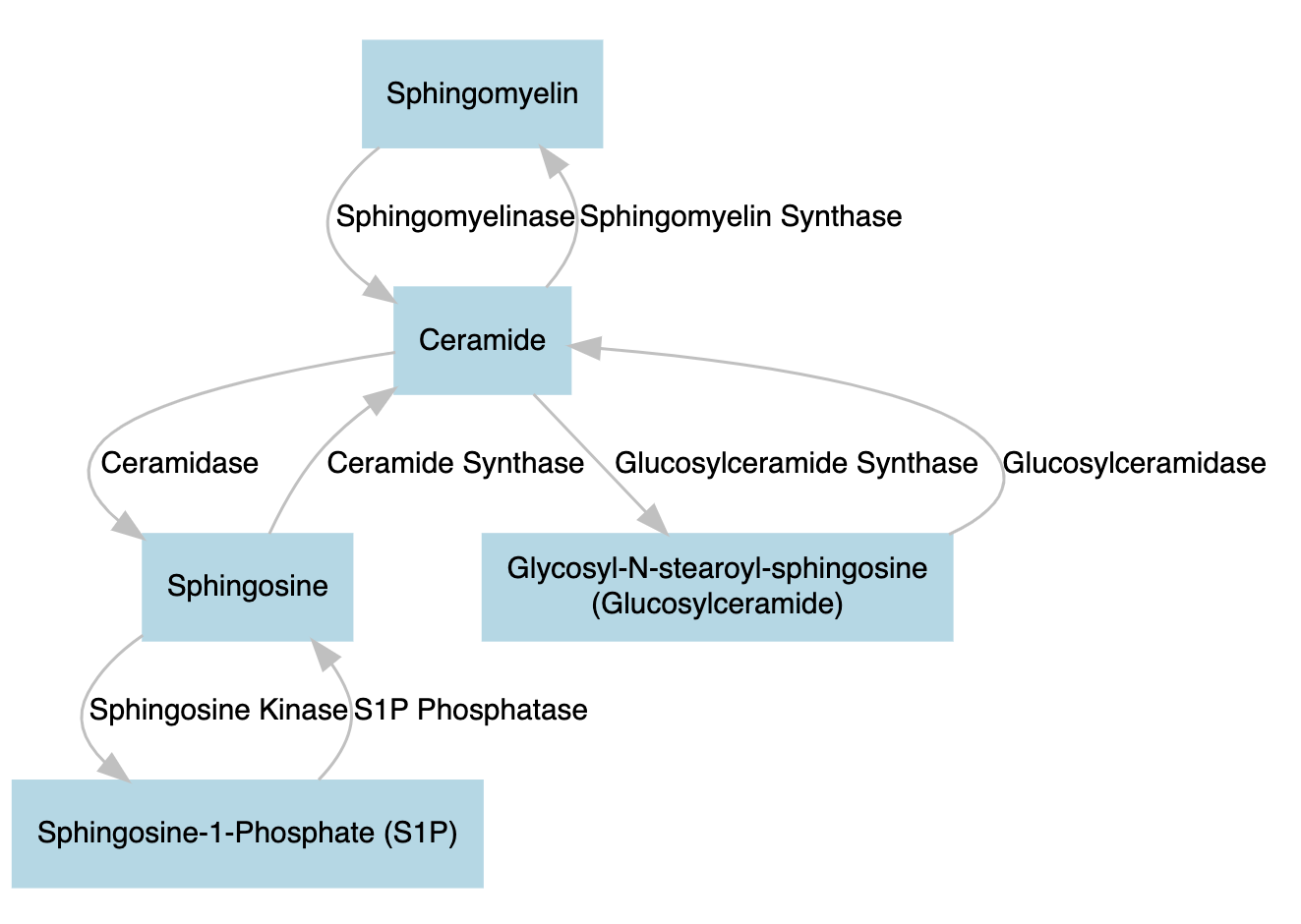

Supplement: Supplementary file 1 — Additional file 1. S1: Description of genetic correlations between neuropsychiatric disorders; Fig S2: Heatmap of genetic correlations between neuropsychiatric disorders, calculated using linkage disequilibrium score regression (LDSC) within GenomicSEM; Fig. S3: Forest plots of IVW-MR estimates for the effect of significant polygenic metabolites on unadjusted and BMI, waist-to-hip ratio and educational attainment adjusted neuropsychiatric outcomes; Fig S4: Simplified metabolic pathway from linoleic acid (LA) to arachadonic acid (AA) derived from KEGG pathway hsa00591; Fig S5: Simplified metabolic pathway for sphingomyelin and ceramide metabolism from KEGG pathway hsa00600. [file 12916_2025_4129_MOESM1_ESM.docx]
