# Supplementary material for: Evaluating metabolome-wide causal effects on risk for psychiatric and neurodegenerative disorders
Source: BMC Med. 2025 Jun 2;23:326. doi: 10.1186/s12916-025-04129-4 (PMC12131761; doi:10.1186/s12916-025-04129-4)
Supplement: Supplementary file 3 — Additional file 3. [file 12916_2025_4129_MOESM3_ESM.docx]

**STROBE-MR checklist of recommended items to address in reports of Mendelian randomization studies**^1^ ^2^

**This checklist is for the manuscript “Evaluating metabolome-wide causal effects on risk for psychiatric and neurodegenerative disorders”**

| **Item No.** | **Section** | **Checklist item** | **Page No.** | **Relevant text from manuscript** |
| --- | --- | --- | --- | --- |
| 1 | **TITLE and ABSTRACT** | Indicate Mendelian randomization (MR) as the study’s design in the title and/or the abstract if that is a main purpose of the study | 2 | Here, we used Mendelian randomisation (MR) to test the causal effects of ~1000 plasma metabolites and ~300 metabolite ratios on anxiety, bipolar disorder, depression, schizophrenia, amyotrophic lateral sclerosis, Alzheimer’s disease, Parkinson’s disease and multiple sclerosis |
|  | **INTRODUCTION** |  |  |  |
| 2 | **Background** | Explain the scientific background and rationale for the reported study. What is the exposure? Is a potential causal relationship between exposure and outcome plausible? Justify why MR is a helpful method to address the study question | 3 | Similar metabolite groups, such as ceramides, are indicated to play a role in both psychiatric and neurodegenerative disorders [23–27]. However, observational studies may be confounded by reverse causation[28]. Importantly for psychiatric and neurodegenerative disorders, the metabolome can be perturbed by disease pathology and/or pharmaceutical interventions[29–33]. As such, following onset and treatment discerning the direction of effect and identifying causal metabolite targets is challenging. These limitations can be addressed using Mendelian randomisation (MR), a statistical framework that leverages genetic variants as instrumental variables (IVs) to proxy an exposure and infer its causal effect on an outcome of interest[34] |
| 3 | **Objectives** | State specific objectives clearly, including pre-specified causal hypotheses (if any). State that MR is a method that, under specific assumptions, intends to estimate causal effects |  | See above, hypothesis free metabolome wide approach |
|  | **METHODS** |  |  |  |
| 4 | **Study design and data sources** | Present key elements of the study design early in the article. Consider including a table listing sources of data for all phases of the study. For each data source contributing to the analysis, describe the following: | 5/30 | Study design illustrated in flowchart for all analyses (Figure 1) |
|  | a) | Setting: Describe the study design and the underlying population, if possible. Describe the setting, locations, and relevant dates, including periods of recruitment, exposure, follow-up, and data collection, when available. | 5 | An overview of the GWAS summary statistics is shown in Table 1. These consisted of individuals of European ancestry only. |
|  | b) | Participants: Give the eligibility criteria, and the sources and methods of selection of participants. Report the sample size, and whether any power or sample size calculations were carried out prior to the main analysis | 5 | See above.   +  Effective sample sizes were calculated as per Grotzinger et al.[57], providing comparable sample size estimates representing equivalently powered GWAS with a 1:1 case/control ratio. |
|  | c) | Describe measurement, quality control and selection of genetic variants | 6-7 | See sections on GWAS standardisation, metabolite instrument selection and MR analyses |
|  | d) | For each exposure, outcome, and other relevant variables, describe methods of assessment and diagnostic criteria for diseases | - |  |
|  | e) | Provide details of ethics committee approval and participant informed consent, if relevant | 20-21 | This research has been conducted using the UK Biobank Resource under Application Number 82877. Ethical approval for the UK Biobank study has been granted by the National Information Governance Board for Health and Social Care and the NHS North West Multicentre Research Ethics Committee (11/NW/0382). Written informed consent was obtained from all participants by the UK Biobank. We thank the UK Biobank Team for collecting the data and making it available. We also thank the UK Biobank participants. |
| 5 | **Assumptions** | Explicitly state the three core IV assumptions for the main analysis (relevance, independence and exclusion restriction) as well assumptions for any additional or sensitivity analysis | 5/30 | Assumptions are illustrated graphically in Figure 1 and stated explicitly in the figure legend |
| 6 | **Statistical methods: main analysis** | Describe statistical methods and statistics used | 6-7 | See section on MR analyses |
|  | a) | Describe how quantitative variables were handled in the analyses (i.e., scale, units, model) | - |  |
|  | b) | Describe how genetic variants were handled in the analyses and, if applicable, how their weights were selected | 6 | Independent instrumental variables (IVs) were initially selected at genome-wide significance (p ≤ 5 x 10-8), clumping at an r2 threshold of 0.001 within 10,000 kb of the lead variants… Where less than five IVs were available for any given exposure, a lower p-value threshold of 5 x 10-6 was used for IV selection as per previous analyses64. If less than 5 IVs were available at the 5 x 10-6 threshold, this metabolite was excluded. Where an IV was not available for the outcome, proxy IVs (i.e., variants in linkage disequilibrium (LD) with the original instrument at r2 > 0.8) were identified using snappy (v.1.0) (https://gitlab.com/richards-lab/vince.forgetta/snappy) and same reference panel. Proxies were selected using the highest r2 value and closest genomic position. Prior to analysis, exposure and outcome were harmonised to the same effect allele, and strand ambiguous palindromic variants (MAF > 0.42) dropped. Instrument strength was assessed via their F-statistic (βexposure2/SEexposure2), with weak instruments (F-statistic < 10) excluded. If less than 5 IVs remained, the metabolite was again excluded. Instrument measurement error was assessed with the I2G-X statistic (< 0.9 suggestive of measurement error)65. |
|  | c) | Describe the MR estimator (e.g. two-stage least squares, Wald ratio) and related statistics. Detail the included covariates and, in case of two-sample MR, whether the same covariate set was used for adjustment in the two samples | 7 | We conducted the primary analyses using inverse variance weighted MR (IVW-MR) with multiplicative random effects as recommended, based on Wald ratio estimates |
|  | d) | Explain how missing data were addressed | 6 | Where an IV was not available for the outcome, proxy IVs (i.e., variants in linkage disequilibrium (LD) with the original instrument at r2 > 0.8) were identified using snappy (v.1.0) (https://gitlab.com/richards-lab/vince.forgetta/snappy) and same reference panel. |
|  | e) | If applicable, indicate how multiple testing was addressed | 7 | If the IVW-MR estimate was statistically significant after Benjamini-Hochberg false discovery rate (FDR) correction67 (pFDR ≤ 0.05, corrected for total number of tests specific to each outcome) |
| 7 | **Assessment of assumptions** | Describe any methods or prior knowledge used to assess the assumptions or justify their validity | 7 | six sensitivity analyses were conducted to assess its robustness to violations of the pleiotropy assumption and instrument validity. We implemented methods that assume a majority valid instruments (weighted median[68]; penalised weighted median[69]), plurality valid instruments (constrained maximum likelihood (cML)[70]; contamination mixture model MR (MR-ContMix)[71]), and that exclude invalid outliers (MR-Lasso[72]). Additionally, we used MR-Egger[73], which gives consistent causal estimates even if all instruments are not valid, provided pleiotropic effects are not correlated with variant-exposure associations. This is known as the Instrument Strength Independent of Direct Effect (InSIDE) assumption and is MR-Egger-specific. |
| 8 | **Sensitivity analyses and additional analyses** | Describe any sensitivity analyses or additional analyses performed (e.g. comparison of effect estimates from different approaches, independent replication, bias analytic techniques, validation of instruments, simulations) | 9 | To pass sensitivity criteria, all sensitivity analysis point estimates needed to be directionally concordant with the IVW estimate and the majority (≥ 4) statistically significant at p ≤ 0.05. Where a metabolite passed sensitivity criteria, we performed reverse IVW-MR as above with the outcome as the exposure. |
| 9 | **Software and pre-registration** |  |  |  |
|  | a) | Name statistical software and package(s), including version and settings used | 7 | We conducted the primary analyses using inverse variance weighted MR (IVW-MR) with multiplicative random effects as recommended66 using the TwoSampleMR package (v.0.5.6). […]MR-ContMix and MR-Lasso were conducted using the MendelianRandomisation package (v.0.9.0), while MR-cML was conducted using the MRcML package (v.0.0.0.9). |
|  | b) | State whether the study protocol and details were pre-registered (as well as when and where) | - | Not pre-registered |
|  | **RESULTS** |  |  |  |
| 10 | **Descriptive data** |  |  |  |
|  | a) | Report the numbers of individuals at each stage of included studies and reasons for exclusion. Consider use of a flow diagram | 5/30 | Flow diagram in figure 1 |
|  | b) | Report summary statistics for phenotypic exposure(s), outcome(s), and other relevant variables (e.g. means, SDs, proportions) | 8-9 | For polygenic score follow-up analyses:  Sample sizes ranged from 140,154 to 356,763, depending on the disorder, with case numbers ranging from 600 (ALS) to 46,900 (DEP) (mean age range = 56.65-64.13; %male range = 46.32-49.35). |
|  | c) | If the data sources include meta-analyses of previous studies, provide the assessments of heterogeneity across these studies | - |  |
|  | d) | For two-sample MR:  i.  Provide justification of the similarity of the genetic variant-exposure associations between the exposure and outcome samples  ii.  Provide information on the number of individuals who overlap between the exposure and outcome studies | - | No overlap |
| 11 | **Main results** |  |  |  |
|  | a) | Report the associations between genetic variant and exposure, and between genetic variant and outcome, preferably on an interpretable scale | - |  |
|  | b) | Report MR estimates of the relationship between exposure and outcome, and the measures of uncertainty from the MR analysis, on an interpretable scale, such as odds ratio or relative risk per SD difference | All results section | MR estimates reported in odds ratio and 95%CI |
|  | c) | If relevant, consider translating estimates of relative risk into absolute risk for a meaningful time period | - |  |
|  | d) | Consider plots to visualize results (e.g. forest plot, scatterplot of associations between genetic variants and outcome versus between genetic variants and exposure) | 12,13 | Forest plots for significant IVW-MR estimates included in Figure 2 and 3 |
| 12 | **Assessment of assumptions** |  |  |  |
|  | a) | Report the assessment of the validity of the assumptions | 10 onwards | Instrument strength reported, sensitivity test criteria pass indicates robustness to pleiotropy violations |
|  | b) | Report any additional statistics (e.g., assessments of heterogeneity across genetic variants, such as *I^2^*, Q statistic or E-value) | 10 | For single instruments, F-statistics ranged from 10.11 to 5625.88 (mean per-test F-statistics range: 20.77-1163.90). Analyses were thus not impacted by weak instruments. Per-test I2G-X ranged from 0.45 to 1.000. Only 94 tests (0.009%), involving 13 unique metabolites, were conducted with I2G-X < 0.9, suggestive of measurement error. Only 27 tests were conducted using instruments with I2G-X ≤ 0.8, involving six metabolites |
| 13 | **Sensitivity analyses and additional analyses** |  |  |  |
|  | a) | Report any sensitivity analyses to assess the robustness of the main results to violations of the assumptions | 11 | Primary IVW-MR analyses identified 138 causal effects involving 113 unique metabolites after outcome-specific FDR correction (Supplementary Table 4; Figure 2a). Of these, 85 metabolite-outcome pairs passed our sensitivity criteria |
|  | b) | Report results from other sensitivity analyses or additional analyses | - | See supplementary table 2 |
|  | c) | Report any assessment of direction of causal relationship (e.g., bidirectional MR) | 11 | No reverse effects were detected for these metabolite-outcome pairs after FDR-correction (Supplementary Table 6). |
|  | d) | When relevant, report and compare with estimates from non-MR analyses | 13-14 | Nominal associations were observed between depression and the PGS for sphingomyelin (d18:2/23:0, d18:1/23:1, d17:1/24:1) and 1-(1-enyl-palmitoyl)-2-arachidonoyl-GPC (P-16:0/20:4) with identical effect sizes (OR[95%CI] = 0.989 [0.979-0.998], p-value range = 2.131 x 10-2 - 2.517 x 10-2) and between the PGS for leucine and PD (OR [95%CI] = 0.962 [0.93- 0.996, p-value = 2.814 x 10-2). These effects were directionally consistent with the MR estimates |
|  | e) | Consider additional plots to visualize results (e.g., leave-one-out analyses) | - |  |
|  | **DISCUSSION** |  |  |  |
| 14 | **Key results** | Summarize key results with reference to study objectives | 17 |  |
| 15 | **Limitations** | Discuss limitations of the study, taking into account the validity of the IV assumptions, other sources of potential bias, and imprecision. Discuss both direction and magnitude of any potential bias and any efforts to address them | 20 | This study used data from individuals of European ancestry only and as such may lack generalisability. Further, as previously noted79, although the exclusion of the APOE region from the AD analyses is necessary to avoid violating MR assumptions, this may result in false negatives due to the regions known role in lipid metabolism126. Conversely, we can have greater confidence that the metabolites identified here are not confounded by APOE effects. Given the importance of this region in AD and lipid metabolism, it deserves specific focus in future metabolomic work. Further, MR requires that several assumptions – such the availability of suitable instruments and absence of pleiotropic effects – be met to provide reliable results. Although we mitigate against these by including only strong instruments (F-statistic ≥ 10) and using a robust sensitivity criterion to delineate causal metabolites, results should be interpreted with caution prior to further triangulation. |
| 16 | **Interpretation** |  |  |  |
|  | a) | Meaning: Give a cautious overall interpretation of results in the context of their limitations and in comparison with other studies | 17 | See full discussion section, also on 20:   Although we mitigate against these by including only strong instruments (F-statistic ≥ 10) and using a robust sensitivity criterion to delineate causal metabolites, results should be interpreted with caution prior to further triangulation. |
|  | b) | Mechanism: Discuss underlying biological mechanisms that could drive a potential causal relationship between the investigated exposure and the outcome, and whether the gene-environment equivalence assumption is reasonable. Use causal language carefully, clarifying that IV estimates may provide causal effects only under certain assumptions | 17 | See full discussion section |
|  | c) | Clinical relevance: Discuss whether the results have clinical or public policy relevance, and to what extent they inform effect sizes of possible interventions | 17 | See full discussion section |
| 17 | **Generalizability** | Discuss the generalizability of the study results (a) to other populations, (b) across other exposure periods/timings, and (c) across other levels of exposure | 20 | Also in limitations |
|  | **OTHER INFORMATION** |  |  |  |
| 18 | **Funding** | Describe sources of funding and the role of funders in the present study and, if applicable, sources of funding for the databases and original study or studies on which the present study is based | 20 | Funding and acknowledgements  LG is funded by the King’s College London DRIVE-Health Centre for Doctoral Training and the Perron Institute for Neurological and Translational Science. JM is supported by the King’s Prize Fellowship. This study has been partly delivered through the National Institute for Health and Care Research (NIHR) Maudsley Biomedical Research Centre (BRC). PP is funded by an Alzheimer’s Research UK Senior Research Fellowship. |
| 19 | **Data and data sharing** | Provide the data used to perform all analyses or report where and how the data can be accessed, and reference these sources in the article. Provide the statistical code needed to reproduce the results in the article, or report whether the code is publicly accessible and if so, where | 21-22 | Data is available on reasonable request from the UK Biobank (https://www.ukbiobank.ac.uk/learn-more-about-uk-biobank/contact-us).  Summary statistics from FinnGen are available online (https://www.finngen.fi/en/access_results) and from the Million Veterans Project via dbGaP request (https://www.ncbi.nlm.nih.gov/projects/gap/cgi-bin/study.cgi?studyid=phs001672.v1.p1).  GWAS summary statistics from the Psychiatric Genomics Consortium (PGC) for Alzheimer’s disease, anxiety, bipolar disorder, depression and schizophrenia are available from their website (https://pgc.unc.edu/for-researchers/download-results/). Summary statistics for anxiety from iPSYCH are also available online (https://ipsych.dk/en/research/downloads). For Parkinson’s disease and ALS, summary statistics are available on GWAS Catalog at https://www.ebi.ac.uk/gwas/studies/GCST009325 and https://www.ebi.ac.uk/gwas/studies/GCST90027164 respectively. For multiple sclerosis, summary statistics are available though the IEU Open GWAS Project (https://gwas.mrcieu.ac.uk/datasets/ieu-b-18/). Summary statistics for metabolites measured from Chen et al.58 are available on GWAS catalog (https://www.ebi.ac.uk/gwas/studies/) under accession numbers GCST90199621-90201020. Access to summary statistics for the metabolites measured by Hysi et al.59 were made available following request to the original authors. |
| 20 | **Conflicts of Interest** | All authors should declare all potential conflicts of interest | 22 | CML is on the Scientific Advisory Board of Myriad Neuroscience and has received honoraria for consultancy from UCB. C.L.-Q. has received consultancy fees from Pfizer. C.L.-Q. has received honoraria, travel or speakers’ fees from Biogen and research funds from Pfizer and Novo Nordisk. C.L.-Q. is the director of the company BrainLogia. All other authors declare no competing interests. |

This checklist is copyrighted by the Equator Network under the Creative Commons Attribution 3.0 Unported (CC BY 3.0) license.

1. Skrivankova VW, Richmond RC, Woolf BAR, Yarmolinsky J, Davies NM, Swanson SA, et al. Strengthening the Reporting of Observational Studies in Epidemiology using Mendelian Randomization (STROBE-MR) Statement. JAMA. 2021;under review.

2. Skrivankova VW, Richmond RC, Woolf BAR, Davies NM, Swanson SA, VanderWeele TJ, et al. Strengthening the Reporting of Observational Studies in Epidemiology using Mendelian Randomisation (STROBE-MR): Explanation and Elaboration. BMJ. 2021;375:n2233.
